# Acteoside protects podocyte against apoptosis through regulating AKT/GSK-3β signaling pathway in db/db mice

**DOI:** 10.1186/s12902-023-01483-3

**Published:** 2023-10-23

**Authors:** Xiaoya Li, Zhilong Liu, Zhixiu He, Xiaocheng Wang, Rongshan Li, Junwei Wang, Guiqiao Ma, Peipei Zhang, Chanjuan Ma

**Affiliations:** 1 Department of Nephrology, Shanxi Provincial People’s Hospital, Taiyuan, China; 2 Department of Nephrology, The Fifth Clinical Medical College of Shanxi Medical University, Fifth Hospital of Shanxi Medical University, Taiyuan, China; 3 Shanxi Provincial Key Laboratory of Kidney Disease, Shanxi Provincial People’s Hospital, Taiyuan, China; 4 The Third Clinical College, Shanxi University of Chinese Medicine, Jinzhong, China; 5 Department of Medical Record & Statistics, Shanxi Provincial People’s Hospital, Taiyuan, China

**Keywords:** Diabetic Kidney Disease (DKD), Acteoside, Podocyte, Apoptosis, AKT/GSK-3β signaling pathway

## Abstract

**Background:**

Podocyte apoptosis is one of the important pathological mechanisms of diabetic kidney disease (DKD). Acteoside (Act), a major active component of Rehmannia glutinosa leaves total glycoside, has a strong renoprotective action. Our study aims to demonstrate Act’s renoprotective actions in db/db mice.

**Methods:**

We adopted C57BLKS/J db/db mice as DKD animal models. After 8 weeks of Act administration, the 24-hour urine albumin, renal function index, and blood lipid levels were quantified using matching kits. Renal pathology was evaluated by HE and PAS staining. The podocyte damage and apoptosis-related signaling pathway were observed by using immunohistochemistry, western blot, and TUNEL staining.

**Results:**

The albuminuria of db/db mice was reduced from 391 ug/24 h to 152 ug/24 h, and renal pathology changes were alleviated after Act administration. The western blot and immunohistochemistry showed that Act treatment upregulated the synaptopodin and podocin expression compared with db/db mice, while the TUNEL staining indicated podocyte apoptosis was inhibited. The B-cell lymphoma-2 (Bcl-2) level was upregulated in the Act group, but cleaved caspase-3 and Bcl-2 associated X protein (Bax) expression declined, while the protein kinase B/glycogen synthase kinase-3β (AKT/GSK-3β) signaling pathway was repressed.

**Conclusions:**

By inhibiting the AKT/GSK-3β signaling pathway, Act protected podocytes from apoptosis, decreasing the urine albumin of db/db mice and delaying the course of DKD.

**Supplementary Information:**

The online version contains supplementary material available at 10.1186/s12902-023-01483-3.

## Introduction

Diabetic kidney disease (DKD) is regarded as one of the most devastating consequences of diabetes mellitus (DM). Approximately 50% of DKD patients will eventually advance to end-stage renal disease (ESRD) [1, 2]. DKD is now the main cause of chronic kidney disease (CKD) in China, having exceeded all other types of glomerulonephritis-related CKD [3]. DKD progresses to glomerular hyperfiltration, increasing proteinuria, decreasing glomerular filtration rate, and ultimately lead to dialysis or death from cardiovascular events.

The main pathological features of DKD are glomerular mesangial cell proliferation, glomerular basement membrane thickening, podocyte disappearance, and extracellular matrix (ECM) accumulation [1]. Podocytes are indispensable for the glomerular filtration membrane, considered as an essential role in the development of proteinuria [4]. Therefore, podocyte injury is considered to be one of the reliable factors for albuminuria formation and DKD progression [5–7]. Podocyte apoptosis is believed to be the primary cause of podocyte damage, leading to the loss of podocyte marker proteins such as synaptopodin and podocin [8, 9].

AKT (protein kinase B) is involved in cell development and apoptosis [10, 11]. Relevant studies have established that the AKT-related signaling pathway is connected with podocytes and proximal renal tubular epithelial cell death under high glucose conditions [12–16]. Glycogen synthase kinase-3β (GSK-3β) participates in cell differentiation, proliferation, survival, and apoptosis [17, 18]. Several researchers had investigated that AKT represses GSK-3β activity via phosphorylating GSK-3β (ser-9) in a mouse model of T2DM and high glucose conditions, promoting podocyte apoptosis, inhibiting autophagy, and aggravating renal damage [18–20]. Therefore, we hypothesize that AKT/GSK-3β signaling pathway acts a crucial role in regulating podocyte apoptosis.

In recent years, it has been found that traditional Chinese medicine (TCM) plays a very good auxiliary and effective therapeutic role in the prevention and treatment of DKD [21–23]. How to better utilize TCM in clinical to postpone the progression of DKD and improve the quality of patient’s life has become a hot issue. Rehmannia glutinosa leaves total glycoside (DHY) was extracted from Rehmannia glutinosa leaf [24, 25]. Various kidney diseases, including DKD, have been treated with DHY. Acteoside (Act) is one of the primary components of DHY. Under high glucose circumstances, DHY and Act were reported to successfully decrease proteinuria in db/db mice, inhibit human renal tubular epithelial cells (HK-2) proliferation, and suppress mesangial cell hypertrophy and fibrosis [24–26]. However, the protective effect of Act on podocytes and its definite mechanism has not been fully explained.

Consequently, our research aimed to explore whether Act inhibits podocyte apoptosis and plays a renoprotective role via repressing AKT/GSK-3β signaling pathway in db/db mice. This study displays a possible scientific theoretical basis for illustrating the mechanism of the Act treatment of DKD.

## Materials and methods

### Experimental animals and ethical statement

We obtained 24 C57BLKS/J db/db male mice aged 5 weeks (SPF grade) and 8 homogeneous wild-type control mice (db/m mice) (SPF grade) from the Nanjing University Model Animal Research Center and housed them at the Experimental Animal Center (SPF grade), Shanxi Provincial People’s Hospital. With a 12 h light/dark cycle, the temperature, and humidity remained consistent at 22 ± 2 °C and 55%, respectively. The animal licensing number was SYXK (Jin 2019-0003). This study was carried out in accordance with NIH guidelines for the care and use of laboratory animals (8th edition, NIH). And the animal research was approved by the Shanxi Provincial People’s Hospital’s ethical committee (No. 363). All applicable international, national, and/or institutional guidelines for the care and use of animals were followed.

Act were a gift from Sichuan Meidakang Pharmaceutical Co. (The lot number of the product is 190602). A week of adaptation was required for all mice. The control group (db/m group) consisted of 8 db/m mice. And we randomly assigned db/db mice to three groups: db/db without medical intervention (db/db group); Act, 40 mg/kg/d (Act group); and captopril, 100 mg/kg/d (Cap group). An equal saline volume was administered in both the db/m and db/db groups, whereas the treatment groups received the corresponding medication dosage intragastrically for 8 weeks. And the body weight of each mouse was monitored weekly. The blood glucose is tested every 2 weeks by tails vein blood collection.

### Urine, blood samples and kidney tissue collection

Metabolic cages were used to collect 24 h urine from all mice. All animals were anesthetized by intraperitoneal injected 1.25% avertin (tribromoethanol, 0.02 mg/g body weight) after 8 h of fasting. The avertin (M2910) were purchased from Nanjing Aibei Biotechnology Co., Ltd. Blood samples were collected and centrifugated (3000 rpm, 4℃, 15 min) to detect various biochemical indicators. All right kidneys were used for the paraffin section, and the left ones were saved at -80℃ for western blot analysis.

### Biochemical assays

We detected Urine protein, serum creatinine (Scr), blood urea nitrogen (BUN), triglyceride (TG), total cholesterol (TC), and low-density lipoprotein (LDL) by using corresponding kits: Urine protein quantitative test kit (Cat. No. C035-2-1), Creatinine (CRE) assay kit (Cat. No. C011-2-1), BUN test kit (Cat. No. C013-2-1), TG test kit (Cat. No. A110-1-1), TC assay kit (Cat. No. A111-1-1), LDL-C test kit (Cat. No. A113-1-1). All the kits were purchased from the Nanjing Institute of Bioengineering.

### Hematoxylin–eosin (HE) staining and periodic acid–Schiff (PAS) staining

The right kidney tissue was processed into 5 μm paraffin section. We performed HE and PAS staining referring to standard protocols. We captured images under a light microscope (Leica, DM 2500, Germany) with a magnification of 400×. The glomerulus morphology was observed under HE staining. At least 20 randomly selected glomerulus per group (n = 3) and the glomerular area was calculated using Image J software v.1.51j8 (Image J software national Institutes of Health, USA). After PAS staining, at least 40 glomeruli per group (n = 3) were randomly selected to assess the severity of glomerulus injury, and the grading was as follows : 0 = normal, Grade 1 < 25%, Grade 2 = 25–50%, Grade 3 = 50–75%, and Grade 4 = 75–100%. Assessment criteria include thickening of the glomerular basement membrane, the glomerular mesangial cells proliferation, and mesangial matrix deposition or Kimmelstiel-Wilson nodule (K-W) formation, podocyte injury. This study used the method of Dusabimana et al. [27]. And the description of the method partly reproduced their wording.

### Western blot analysis

Mice kidney tissue was cut into small pieces and mixed with RIPA (Proteintech, Wuhan, China) buffer mixed with protease inhibitors (Proteintech, Wuhan, China) and phosphatase inhibitors (Proteintech, Wuhan, China) for 30 min, then the mixture was fully cracked under an ultrasonic comminution apparatus. The supernatant was then collected and detected using a BCA kit. Samples containing the same quantity of protein (50 ug protein) were transferred to PVDF membranes after SDS-PAGE separation. Then it was sealed for 90 min with 5% skim milk. The primary antibodies were then incubated overnight at 4℃. The primary antibodies were purchased from Proteintech, China: anti-AKT (60203-2-Ig), anti-p-AKT (Ser 473) (66444-1-Ig), anti-B-cell lymphoma-2 (Bcl-2) (12789-1-AP), anti-Bcl-2 associated X protein (Bax) (50599-2-Ig), anti-podocin (20384-1-AP), anti-synaptopodin (21064-1-AP), anti-cleaved caspase-3(66470-2-Ig), anti-Glyceraldehyde-3-phosphate dehydrogenase (GAPDH) (60004-1-Ig), anti-β-Actin (20536-1-AP). We purchased anti-GSK-3β (sc-377,213) and anti-p-GSK-3β (sc-373,800) from Santa Cruz Biotechnology, USA. The next day, the antigen-antibody reaction was observed by the ECL kit after the second antibodies were incubated including anti-rabbit IgG (SA00001-2, Proteintech, China) and anti-mouse IgG (A0216, Beyotime Institute of Biotechnology, China).

### Immunohistochemical staining analysis

The 3 μm paraffin slides of the kidney were deparaffinized with xylene, hydrated with graded ethanol, and repaired with Ethylene Diamine Tetraacetic Acid (EDTA) for antigen extract. We used 5% goat serum to block the samples after soaking them with 3% hydrogen peroxide for 22 min at room temperature. It was then treated for 35 min with goat anti-rabbit IgG that had been labeled by horseradish peroxidase after being incubated with anti-podocin (1:300) and anti-synaptopodin (1:300) overnight. We adopted light microscopy (Leica, DM 2500, Germany) to capture the images under an optical microscope magnification of 400×. The mean optical density (MOD) was scanned by employing the Image-Pro Plus 5.0.

### Terminal deoxynucleotidyl transferase dUTP nick-end labeling (TUNEL) assay

We performed TUNEL staining to assess the apoptosis level in mice kidney paraffin sections by using CoraLite®594 TUNEL apoptosis detection kit (Proteintech, Wuhan, China) according to the protocols. A fluorescence microscope (Leica, DM 2500, Germany) was adopted to obtain images under an optical microscope magnification of 400×.

### Podocyte culture

The immortalized mouse podocyte cell line mouse podocyte clone 5 (MPC5) was purchased from China Fenghui Biology. Podocytes were cultured in 1640 medium (Gibco, USA) supplemented with 10% Fetal Bovine Serum (FBS) and 1% penicillin and streptomycin, incubated in a CO2 incubator at 37 °C with 5% CO2 for 10–14 days to induce differentiation, and then cultured for 12 h at 37 °C in FBS-free RPMI-1640 medium containing 5.5 mM D-glucose before subsequent experiments. Upon reaching a cell density of 90%, cell digestion and passaging were performed using 0.25% trypsin.

In addition, MPC5 cells were treated with 5.5 mmol/L glucose (normal glucose, NG), 5.5 mmol/L glucose + 34.5 mmol/L mannitol (Man), 40 mmol/L glucose (High glucose, HG), 40 mmol/L glucose + 100 µmol/L Actroside (Act), 40 mmol/L glucose + 100 µmol/L Captopril (Cap) at 37 °C for 48 h.

### Quantitative real-time PCR (qRT-PCR)

After administering drug treatment, total RNA was extracted from cells using the RNAiso Plus (Takara, Taiyuan, China), and the concentration was determined. Subsequently, cDNA synthesis was carried out following the instructions provided with the reverse transcription kit (Takara, Taiyuan, China), using the primer sequences provided in Table 1. And the primer were Synthesized by Shanghai Sangon Biological Engineering Co. The obtained cDNA was used as a template for amplification with the following program: initial denaturation at 95 °C for 10 min, followed by denaturation at 95 °C for 5 s, annealing at 60 °C for 30 s, and 40 cycles of amplification. The expression levels of the target genes were evaluated using the 2^-ΔΔCt method. Quantification of related mRNA level was normalized to β-actin mRNA level.

**Table 1:**
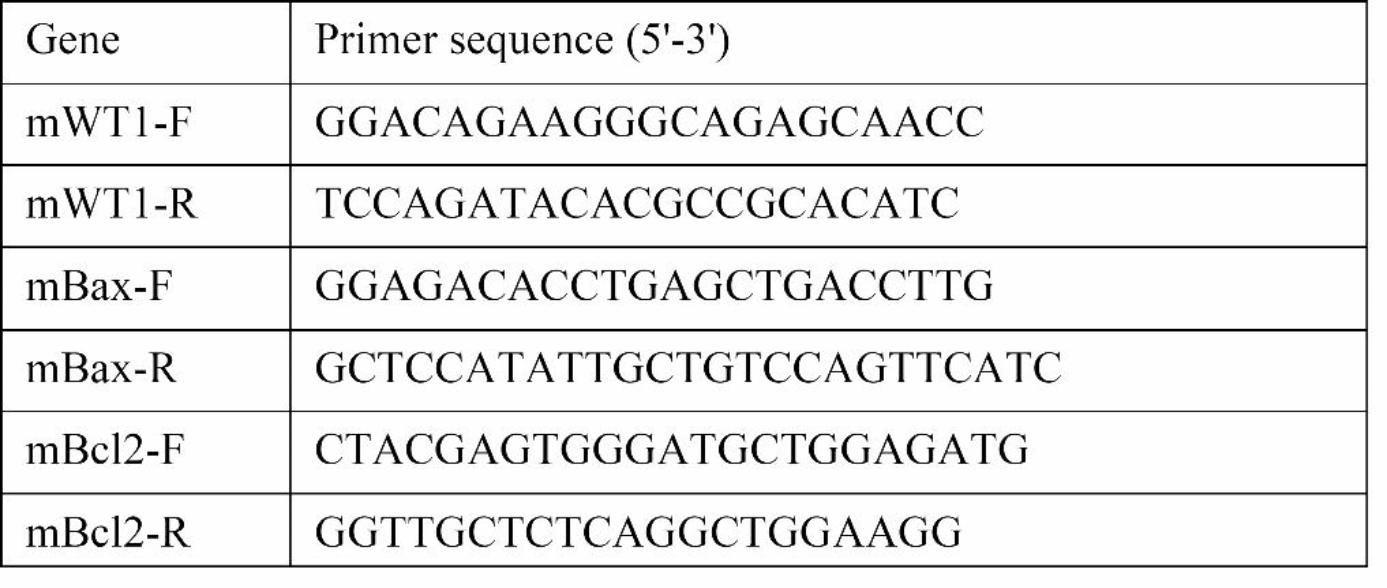
Primers used for qRT-PCR

### Flow cytometry

MPC5 cells were thoroughly washed with PBS and enzymatically dissociated using 0.25% trypsin (without EDTA). Subsequently, the cells were centrifuged at 1000 rpm and the supernatant was carefully discarded. This was followed by two washes with PBS to ensure complete removal of any residual trypsin or debris. The resulting cell suspension, comprising 1 × 10^5^ cells, was once again centrifuged at 1000 rpm for 5 min. After removing the supernatant, 500 µL of binding buffer was added to facilitate subsequent analysis. Furthermore, 5 µL of Annexin V-FITC and 5 µL of propidine iodide (PI) were added to the cell suspension, followed by a 10-minute incubation in a light-protected environment. Thus, the levels of cell apoptosis were assessed through the utilization of flow cytometry.

### Statistical analysis

We used SPSS 26.0 statistical software (SPSS software Inc, IBM Corp, Armonk, New York, USA) to analyze the data by Student’s t-test and one-way ANOVA (LSD or Tamnheini tests) when appropriate. The results were exhibited as the mean ± SD. Paired t-test was used for the urine protein of mice before and after drug intervention. To examine the ranking data, we used the rank sum test. *P* < 0.05 indicated a statistically significant difference.

## Results

### Act decreased albuminuria and improved renal function of db/db mice

**Fig. 1:**
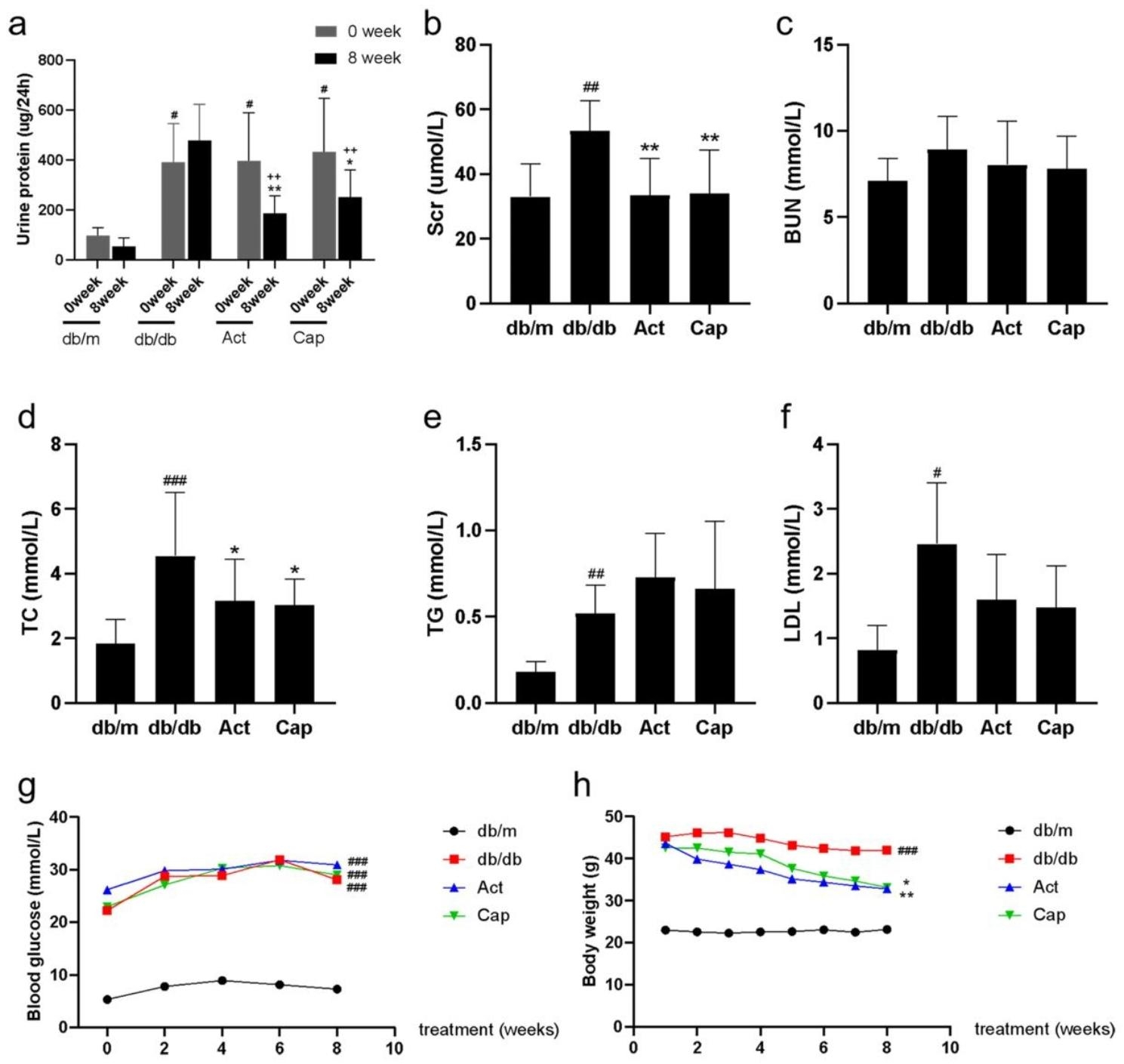
Act decreased albuminuria and improved the renal function of db/db mice. (**a**) The 24-hour albuminuria before and after the administration; (**b-f**) The levels of Scr, BUN, TC, TG, LDL; (**g-h**) The blood glucose and body weight of mice in different times of drug intervention. The results were presented as means ± SD. #*P* < 0.05, ##*P* < 0.01, and ###*P* < 0.001 vs. contemporary db/m group; *P < 0.05, **P < 0.01, and ***P < 0.001 vs. contemporary db/db group; +*P*<0.05, ++*P*<0.01 vs. the mice before experimental treatment

To assess Act’s renoprotective impact on db/db mice, we evaluated urine protein, Scr, BUN, and serum lipid levels. Before treatment, the db/db group, Act group, and Cap group had higher 24-hour urine protein compared with db/m group. Then the albuminuria was dramatically reduced in the Act and Cap groups after 8 weeks of drug intervention compared with the experimental treatment before (Fig. 1a). In comparison to db/m mice, db/db group had higher Scr levels, which were significantly reduced by Act therapy (Fig. 1b). A renal protective effect of Act was observed, although there was no substantial difference in BUN levels among groups (Fig. 1c). Additionally, db/db mice showed increased TC, TG, and LDL levels compared to db/m mice. Act medication considerably dropped the TC level (Fig. 1d), but TG and LDL level differences between the db/db and Act groups were not statistically significant (Fig. 1e-f). Whereas there was no difference between the Act and Cap groups, the blood glucose levels in the db/db group, Act group, and Cap group were all obviously higher than the db/m group (Fig. 1g). We found the body weight of mice in the Act group and Cap group decreased compared with the db/db group after 8 weeks of drug treatment (Fig. 1h). These data confirmed that Act treatment significantly reduced urinary protein, Scr, and TC levels, lowered body weight, and exerted a renoprotective but not hypoglycemic effect in db/db mice.

### Act improved the pathological injury of db/db mice

**Fig. 2:**
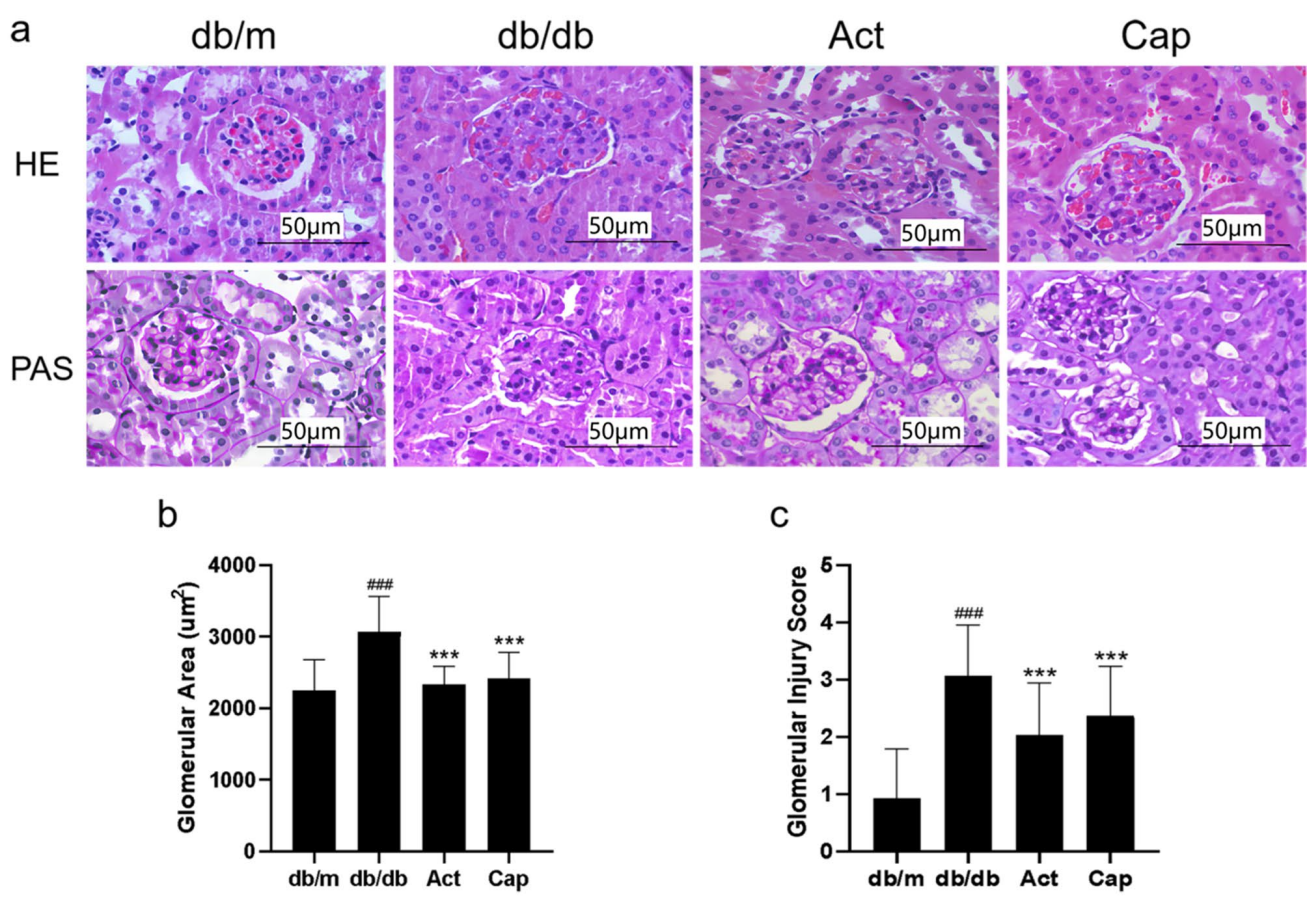
Act improved pathological injury in db/db mice. (**a**) HE and PAS staining analysis of kidney tissues (Scale bar = 50 μm); (**b**) The glomerular area was calculated using Image J software after HE staining; (**c**) The degree of glomerular pathological damage score was performed in PAS staining. The results were presented as means ± SD. #*P* < 0.05, ##*P* < 0.01 and ###*P* < 0.001 vs. db/m group; *P < 0.05, **P < 0.01 and ***P < 0.001 vs. db/db group

Utilizing HE and PAS staining, we were able to see the glomeruli pathology of mice. The HE staining presented that the glomerulus in the db/m group was regular in shape and complete in structure, while the glomerulus in the db/db group was irregular, enlarged volume, and destroyed in capillary loops. The Act and Cap treatment group effectively alleviated the glomerular pathological damage (Fig. 2a). The db/db mice’s glomerular area was larger in contrast to db/m mice, while Act medication substantially lessened the glomerular area swelling (Fig. 2b). Moreover, PAS staining revealed that the number of mesangial cells and mesangial matrix deposition were increased in the db/db mice than in db/m mice, and the basement membrane was thicker (Fig. 2a), likewise, the db/db group had a notable higher glomerular injury score(Fig. 2c). The abovementioned changes were ameliorated after the Act intervention (Fig. 2a-c). Consequently, the Act effectively diminished renal pathological injury of db/db mice.

### Act protected podocytes and inhibited podocytes apoptosis in db/db mice and MPC5 cells

**Fig. 3:**
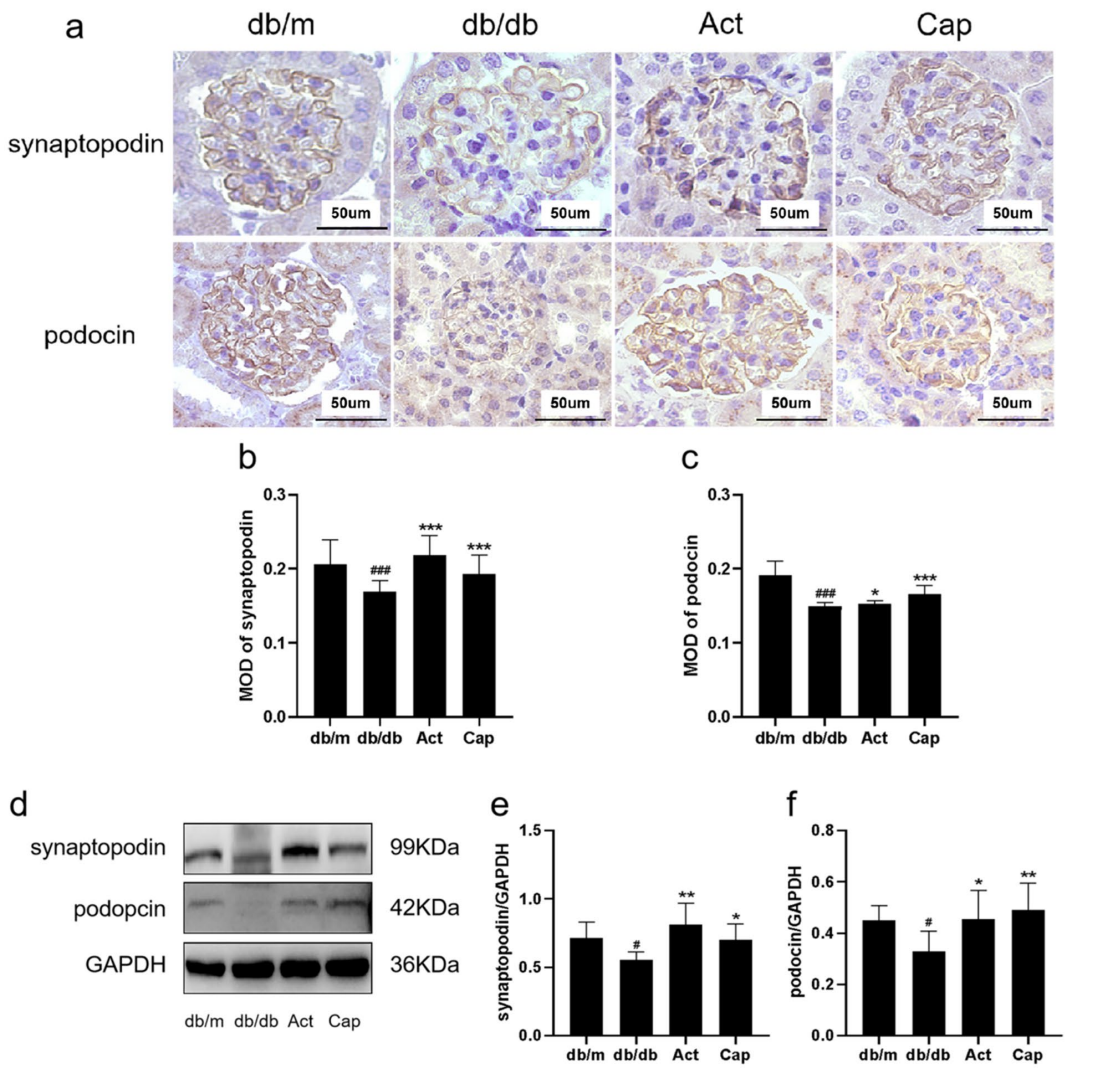
Act protected renal podocyte in db/db mice. (**a-c**) Immunohistochemistry of synaptopodin and podocin in mice (Scale bar = 50 μm); (**d-f**) The western blot consequences of synaptopodin and podocin. The results were shown as means ± SD. #P < 0.05, ##P < 0.01, and ###P < 0.001 vs. db/m group; *P < 0.05, **P < 0.01, and ***P < 0.001 vs. db/db group

**Fig. 4:**
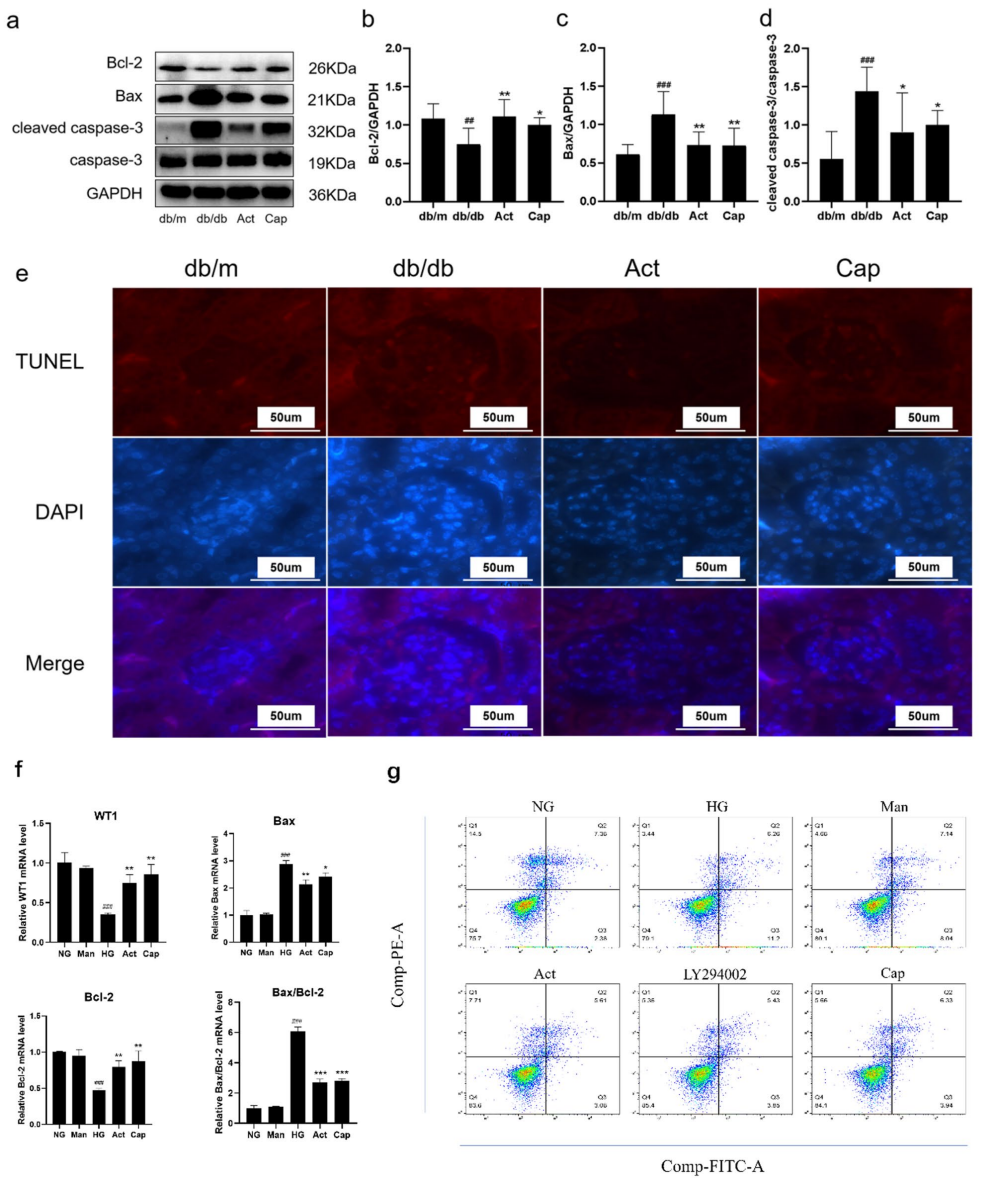
Effects of Act on renal tissue and podocytes. (**a-d**) The Bcl-2, Bax, and cleaved caspase-3 expression in mice kidney tissue; (**e**) TUNEL assay in mice renal tissues (Scale bar = 50 μm); DAPI, blue, apoptotic cell, red. (**f**) Real-time RT-PCR quantitation of WT1、Bax、bcl-2. (**g**) Differences in apoptosis of MPC5 cells between normal glucose, high glucose and Act interventions: Q1 is necrotic cells, Q2 is late apoptotic cells, Q3 is early apoptotic cells and Q4 is the number of viable cells. The data were exhibited as means ± SD. #*P* < 0.05, ##*P* < 0.01, and ###*P* < 0.001 vs. db/m group; *P < 0.05, **P < 0.01, and ***P < 0.001 vs. db/db group

We then determined whether Act exerted a protective effect on podocytes in db/db mice and on MPC5 cells (Figure 3–4). According to the immunohistochemical findings, the synaptopodin and podocin expressions were lower in the db/db mice than in db/m mice, indicating that podocytes in db/db mice had suffered severe damage. While the decrease of synaptopodin and podocin expression was greatly improved by Act therapy (Figur 3a-c). Relative to db/m mice, the western blot analysis revealed that although synaptopodin and podocin expression was noticeably lower in the db/db group, it rose following 8 weeks of Act intervention (Fig. 3d-f). Additionally, it was shown that Act administration reversed the higher Bax and cleaved caspase-3 levels, and restored the lower Bcl-2 level in db/db mice, indicating that Act substantially suppressed the podocyte apoptosis in db/db mice (Fig. 4a-d). In line with the above findings, TUNEL staining revealed that there were more apoptotic regions in db/db mice, while the Act treatment reduced the apoptosis area (Fig. 4e). In comparison with the db/m group, the podocyte apoptosis in the db/db group was noticeably higher. We next further verified whether Act could protect podocytes from apoptosis under high glucose conditions in MPC5 cells. The qPCR results showed that the WT1 and Bcl-2 mRNA levels of MPC5 were significantly down-regulated, Bax mRNA level was up-regulated under high glucose conditions, indicating that apoptosis occurred in the podocytes under hyperglycemic environment, whereas the decrease of the expression of WT1 and bcl-2, and the increase of the expression of Bax were reversed after Act intervention. Meanwhile, the results of flow cytometry further verified that high glucose promotes apoptosis in podocytes, whereas after Act intervention, podocyte apoptosis was reduced, suggesting that Act may protect podocytes from high glucose toxicity through its anti-apoptotic effect. According to the results above, Act protected podocytes against apoptosis in db/db mice and MPC5 cells.

### Act regulated the AKT/GSK-3β signaling pathway in db/db mice

**Fig. 5:**
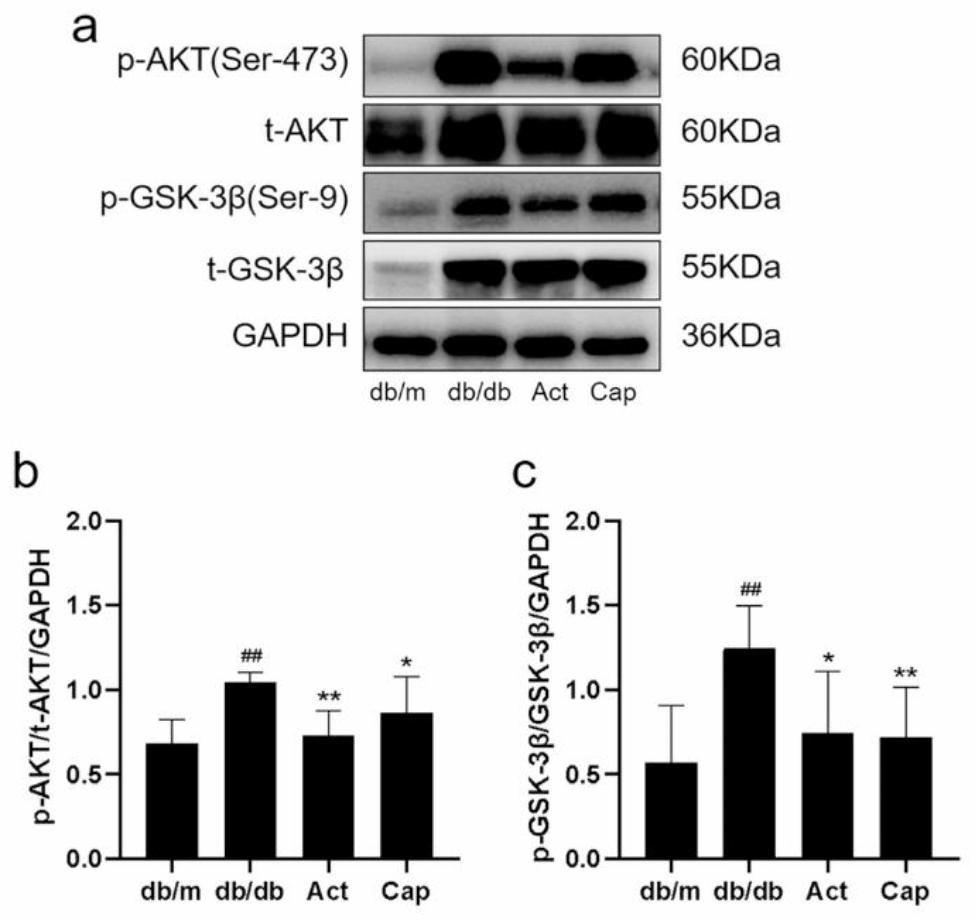
The protective effect of Act on podocytes may correlated with the AKT/GSK-3β signaling pathway inhibitory. (**a-c**) Western blot consequences of p-AKT, p-GSK-3β in mice kidney tissues. The data were exhibited as means ± SD. #*P* < 0.05, ##*P* < 0.01, and ###*P* < 0.001 vs. db/m group; *P < 0.05, **P < 0.01, and ***P < 0.001 vs. db/db group

The AKT/GSK-3β signaling pathway is a typical negatively related signaling pathway regulating tissue apoptosis. According to the western blot outcome, the db/db mice had higher p-AKT and p-GSK-3β (ser-9) expression levels than the db/m mice. However, the p-AKT and p-GSK-3β (ser-9) expression declined with Act and Cap treatment (Fig. 5), which means that AKT/GSK-3β signaling pathway was blocked after Act administration. Given our previous findings, we proposed the hypothesis that Act prevented apoptosis of podocytes and acted as a renoprotective agent in db/db mice by blocking the AKT/GSK-3β signaling pathway.

## Discussion

In db/db mice, we investigated whether Act could protect the kidneys by decreasing podocyte apoptosis. The Act was shown to reduce albuminuria, improve renal dysfunction, and ameliorate renal pathological abnormalities. Then we assessed synaptopodin and podocin expression levels, which were lower in db/db mice, showing that podocytes had been injured. After Act administration, synaptopodin and podocin expression was upregulated, Bcl-2 expression was increased, Bax and cleaved caspase-3 expression were decreased. The results of TUNEL staining demonstrated that Act might reduce kidney apoptosis. Furthermore, we verified by qPCR and flow cytometry in MPC5 cells that high glucose promotes apoptosis in podocytes and that Act intervention could protect podocytes from high glucose damage through its anti-apoptotic effect. We also found that the activity of p-AKT/ p-GSK-3β (Ser-9) was significantly elevated in the kidney tissue of db/db mice compared with db/m mice and decreased after Act drug intervention. This might suggest that Act treatment prevents podocyte apoptosis due to the greatly inhibited activity of p-AKT and p-GSK-3β (Ser-9). Consequently, we hypothesized that Act might reduce podocyte apoptosis and decrease proteinuria by blocking the AKT/GSK-3β signaling pathway to delay the course of DKD in db/db mice.

DHY has a wide range of effects including immunological regulation, inhibition of inflammatory response, and reduction of urinary protein excretion rate [24]. A randomized controlled study found that the combination of Rehmannia glutinosa acteosides and irbesartan was more effective than irbesartan alone in the treatment of chronic glomerulonephritis [28]. According to Wang et al. [29], who studied the interventional effects of Act on HK-2 cells and db/db mice in a high-glucose environment, Act was efficient in reducing urine protein and restoring renal function in db/db mice, which is consistent with our findings. And the specific mechanism may be to suppress inflammation and oxidative stress in HK-2 cells by inhibiting the NADPH/oxidase-TGF-β/Smad signaling pathway, thereby alleviating renal fibrosis and ultimately ameliorating early renal damage in DKD. However, the effects and mechanisms of Act intervention on podocytes have not been explored.

Podocyte apoptosis is one of the major podocytes’ responses to various injuries, which include glomerular hyperfiltration, hypertension, hyperglycemia, and various cytokines and hormones [30]. Podocyte apoptosis plays an essential role in DKD development [5–7, 31]. Accumulating studies suggested that targeting inhibition of podocyte apoptosis could relieve the progression of DKD to some extent [32–34]. Our research indicated that Act upregulated synaptopodin and podocin protein levels, inhibited podocyte apoptosis, reduced urine albumin, and ameliorated renal pathological injury in db/db mice. Similarly in MPC5 cells, the results of qPCR and flow cytometry suggested that high glucose promotes podocyte apoptosis, whereas Act intervention protects podocytes and inhibits the apoptotic activity. Therefore, it is noteworthy that our study demonstrated that Act protected podocytes from apoptosis caused by the diabetic environment, decreased the albuminuria, and protected the renal function, thus delaying the development of DKD in db/db mice.

AKT, as the core part of the PI3K/AKT/mTOR signaling pathway, is essential in animal models of DKD and high glucose-induced podocyte damage [16]. Several studies have observed that AKT was activated in diabetic rats induced by streptozotocin (STZ), podocytes, and proximal tubular epithelial cells under high glucose conditions [35–38], which might be the risk factor for renal fibrosis and DKD progression, and it was consistent with our findings. GSK-3β was identified as one of the downstream targets of AKT, which inhibits GSK-3β activity by phosphorylating GSK-3β (ser-9) [20]. Dusabimana et al. [19] found that AKT was activated in the DKD mouse model and subsequently phosphorylated GSK-3β (Ser-9) which was the inhibitory site of GSK-3β, thereby inhibiting GSK-3β activity. While p-AKT and p-GSK-3β (Ser-9) were significantly inhibited after Geniposide treatment, resulting in decreased proteinuria and podocytes loss, and improved renal function. Xu et al. [13] found that autophagy was inhibited and apoptotic activity significantly increased in human podocytes cultured with high glucose, which may correlate with abnormal AKT/mTOR signaling pathway. In other words, inhibition of the AKT signaling pathway can improve podocytes loss and reduce proteinuria in the progression of DKD. Our findings revealed that p-AKT and p-GSK-3β activities were considerably increased in db/db mice as compared to db/m mice, while Act suppressed the expression of p-AKT and p-GSK-3β, which was consistent with previous findings. As a result, we propose that Act suppressed the AKT/GSK-3β signaling pathway, which in turn reduced podocyte apoptosis, improved glomerular pathology, and eventually delayed the development of DKD in db/db mice.

we investigated the effect of Act on podocyte apoptosis, however, the deficiency of this study is lacking further study on the regulatory effect of Act on AKT/GSK-3β signaling pathway. Thus, more in vitro studies are urgently needed to elucidate the regulatory mechanisms of Act on the AKT/GSK-3β signaling pathway in podocytes.

**Fig. 6:**
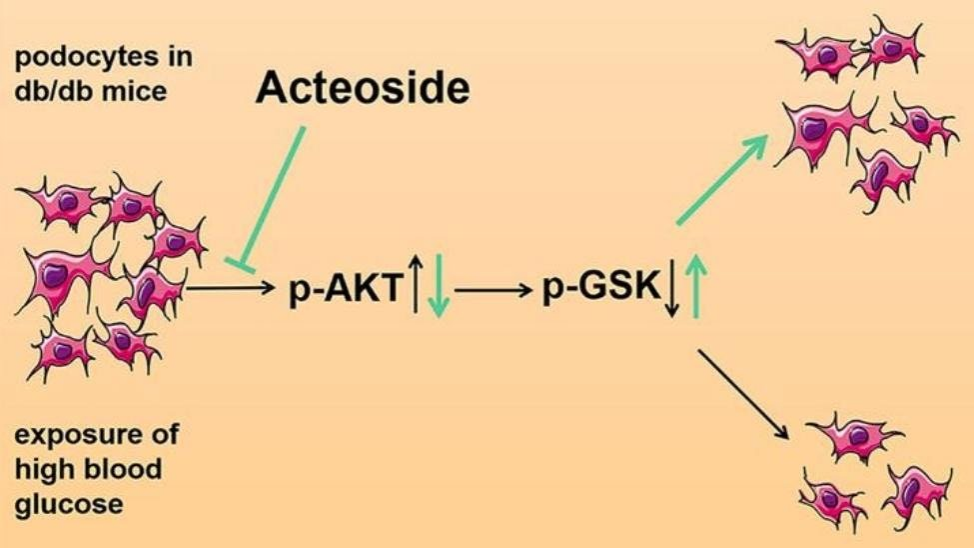
The Act reduced the apoptosis activity of podocytes by blocking the AKT/GSK-3β signaling pathway, thus decreasing the proteinuria of db/db mice, and ameliorating DKD progression

In summary, our investigation elucidated a prospective renoprotective mechanism, whereby Act suppressed the AKT/GSK-3β signaling pathway and reduced podocyte apoptosis in db/db mice, resulted in decreased proteinuria (Fig. 6), indicating that Act might be a potential renoprotective agent by alleviating DKD progression.

### Electronic supplementary material

Below is the link to the electronic supplementary material.

**Figure 3**. Act protected renal podocyte in db/db mice. The western blot consequences of synaptopodin and podocin. The images are typical and representative. **Figure 4**. Effects of Act on renal tissue and podocytes. The Bcl-2, Bax, and cleaved caspase-3 expression in mice kidney tissue. The images are typical and representative. **Figure 5**. The protective effect of Act on podocytes may correlated with the AKT/GSK-3β signaling pathway inhibitory. Western blot consequences of p-AKT, p-GSK-3β in mice kidney tissues. The images are typical and representative.

## Data Availability

The datasets supporting the results of this article are included within the article. Further enquiries can be directed to the corresponding author.

## Abbreviations

Act: Acteoside
AKT: Protein kinase B
Bcl-2: B-cell lymphoma-2
Bax: Bcl-2 associated X protein
BUN: Blood urea nitrogen
CKD: Chronic kidney disease
DHY: Rehmannia glutinosa leaves total glycoside
DKD: Diabetic kidney disease
DM: Diabetes mellitus
ESRD: End-stage renal disease
GSK-3β: Glycogen synthase kinase-3β
HE: Hematoxylin-Eosin staining
LDL: Low-density lipoprotein
PAS: Periodic Acid-Schiff staining
Scr: Serum creatinine
TCM: Traditional Chinese Medicine
TG: Triglyceride
TC: Total cholesterol
TUNEL: TdT-mediated dUTP nick end labeling staining
