# Supplementary material for: Acteoside protects podocyte against apoptosis through regulating AKT/GSK-3β signaling pathway in db/db mice
Source: BMC Endocr Disord. 2023 Oct 23;23:230. doi: 10.1186/s12902-023-01483-3 (PMC10591407; doi:10.1186/s12902-023-01483-3)
Supplement: Supplementary file 1 — Figure 3. Act protected renal podocyte in db/db mice. The western blot consequences of synaptopodin and podocin. The images are typical and representative. Figure 4. Effects of Act on renal tissue and podocytes. The Bcl-2, Bax, and cleaved caspase-3 expression in mice kidney tissue. The images are typical and representative. Figure 5. The protective effect of Act on podocytes may correlated with the AKT/GSK-3β signaling pathway inhibitory. Western blot consequences of p-AKT, p-GSK-3β in mice kidney tissues. The images are typical and representative. [file 12902_2023_1483_MOESM1_ESM.docx]

Gels/blots are used in figure 3

synaptopodin

db/db

db/m

db/db

Act

Cap

Act

db/m

Cap


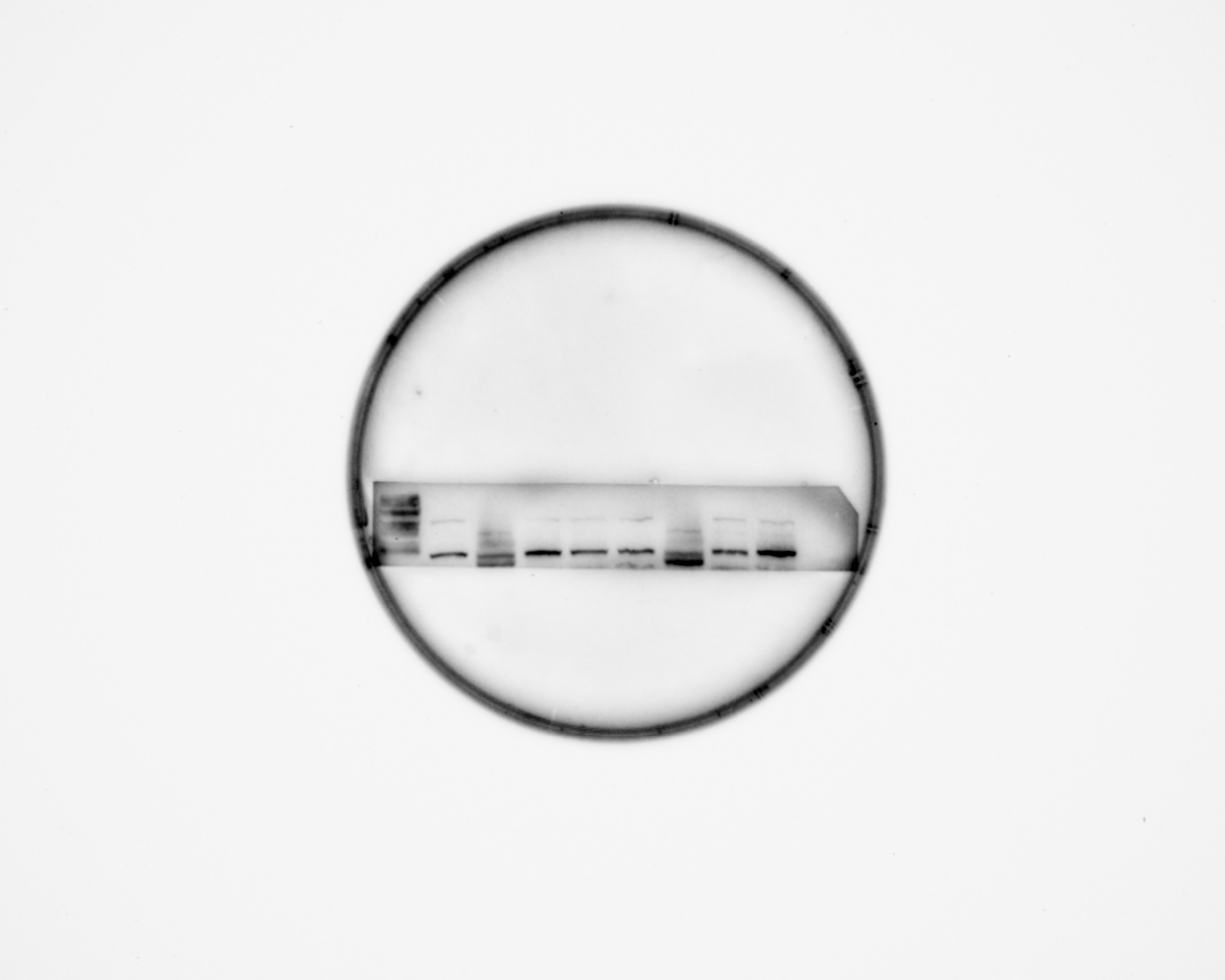


Podocin

Cap

Act

db/db

db/m

db/db

Act

db/m

Cap


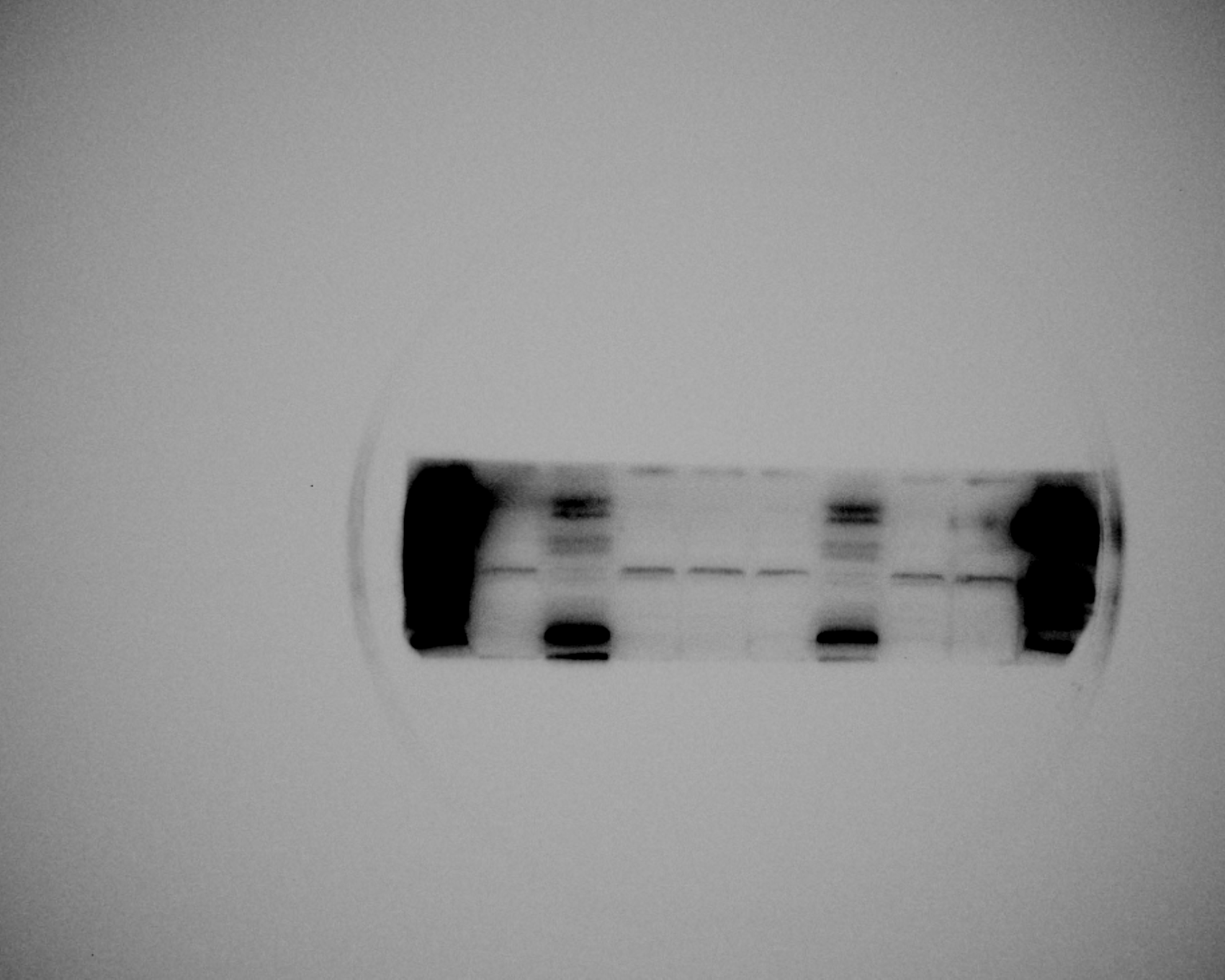


GAPDH

db/db

Act

Act

db/db

db/m

db/m

Cap

Cap


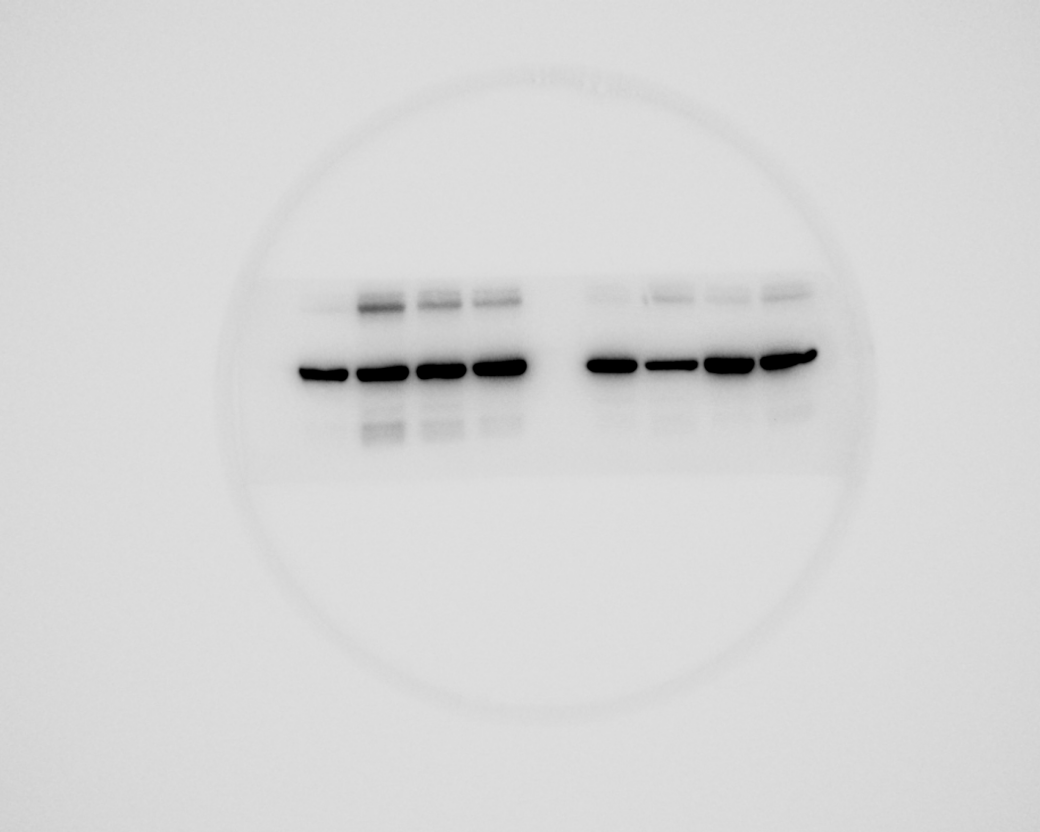


Figure 3. Act protected renal podocyte in db/db mice. The western blot consequences of synaptopodin and podocin. The images are typical and representative.

Gels/blots are used in figure 4

Bcl-2 Bax

db/db

db/m

db/m

Cap

Act

db/db

Act

Cap


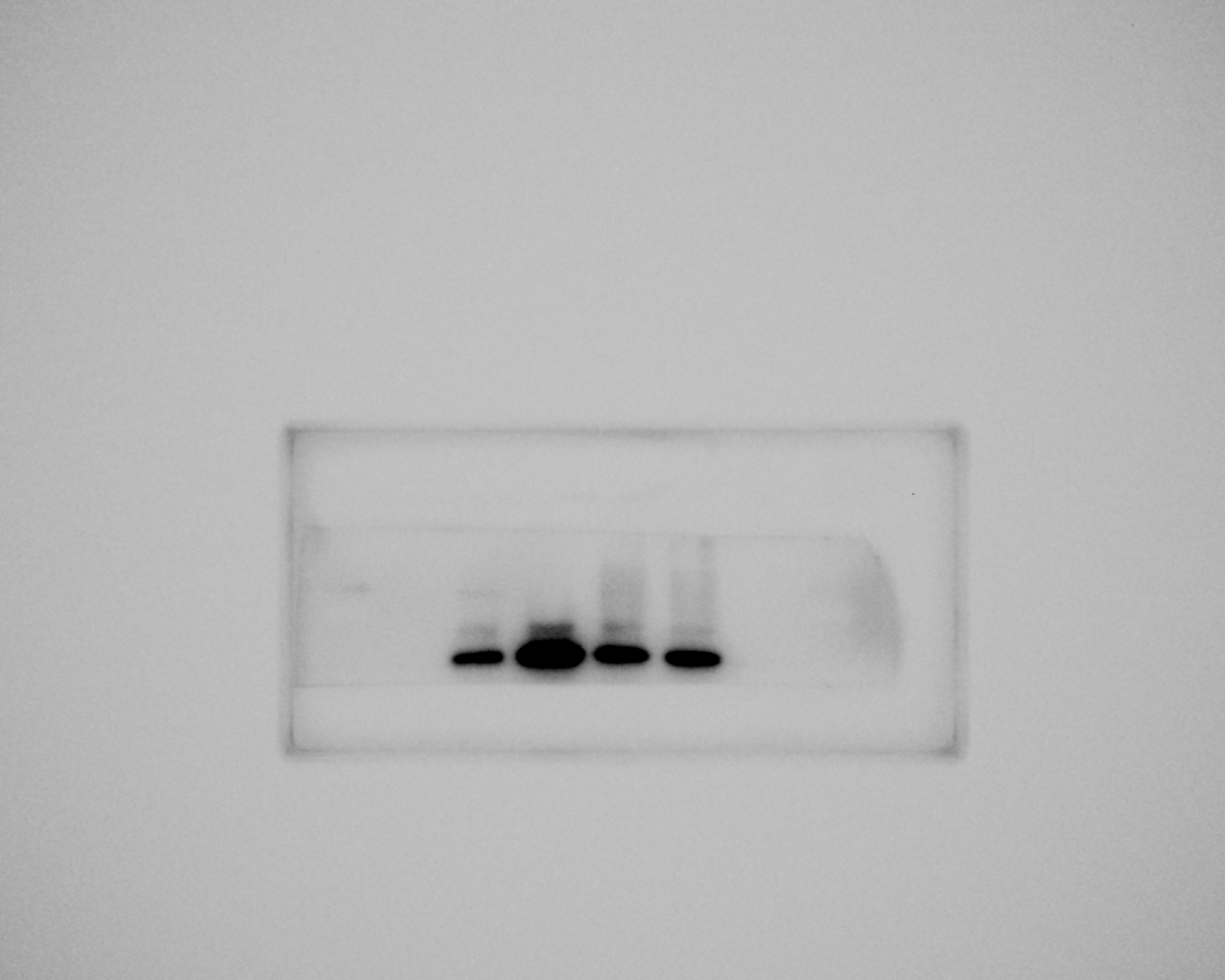

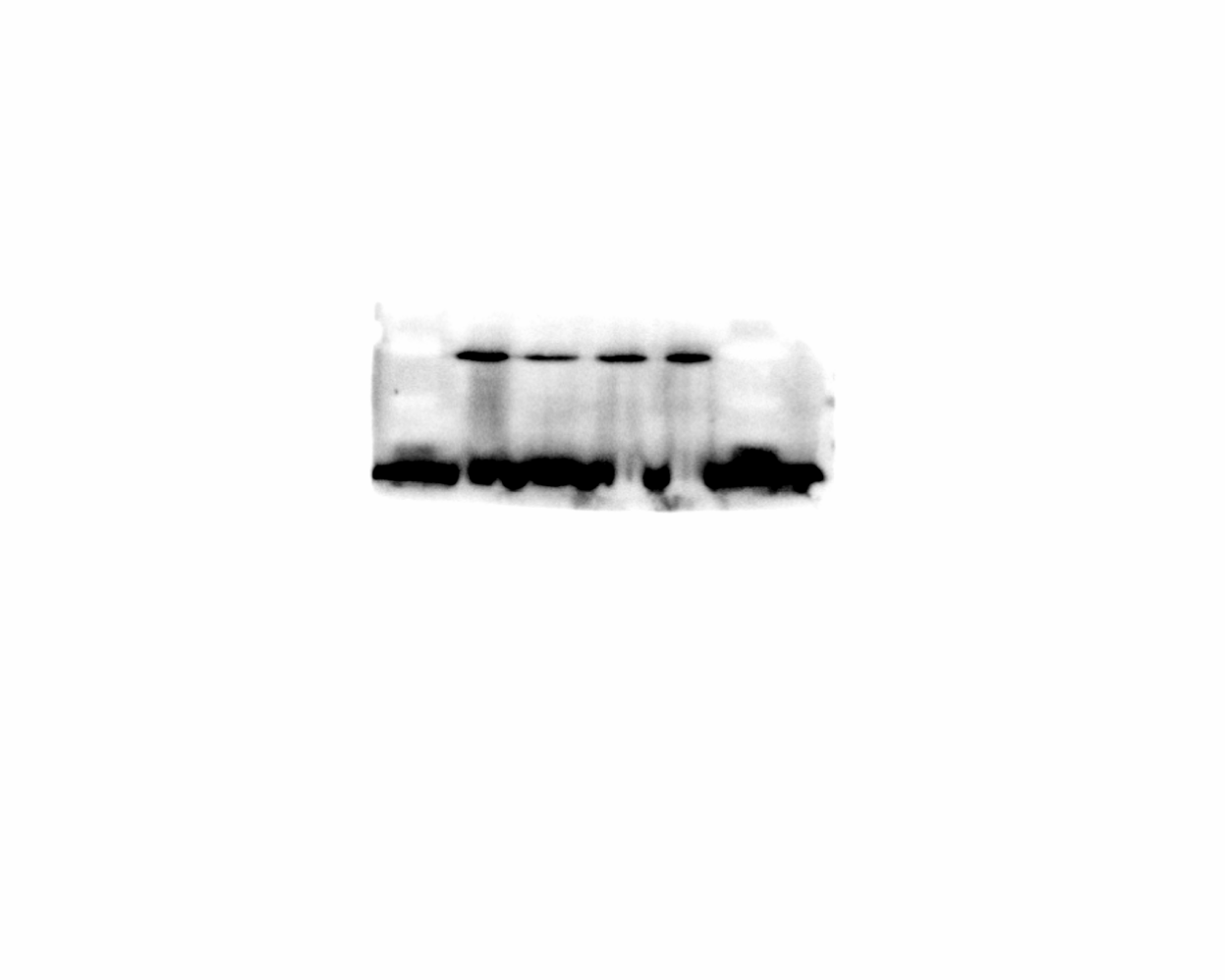


Cleaved casepase-3

Act

Cap

db/m

db/db

db/db

db/m

Act

Cap


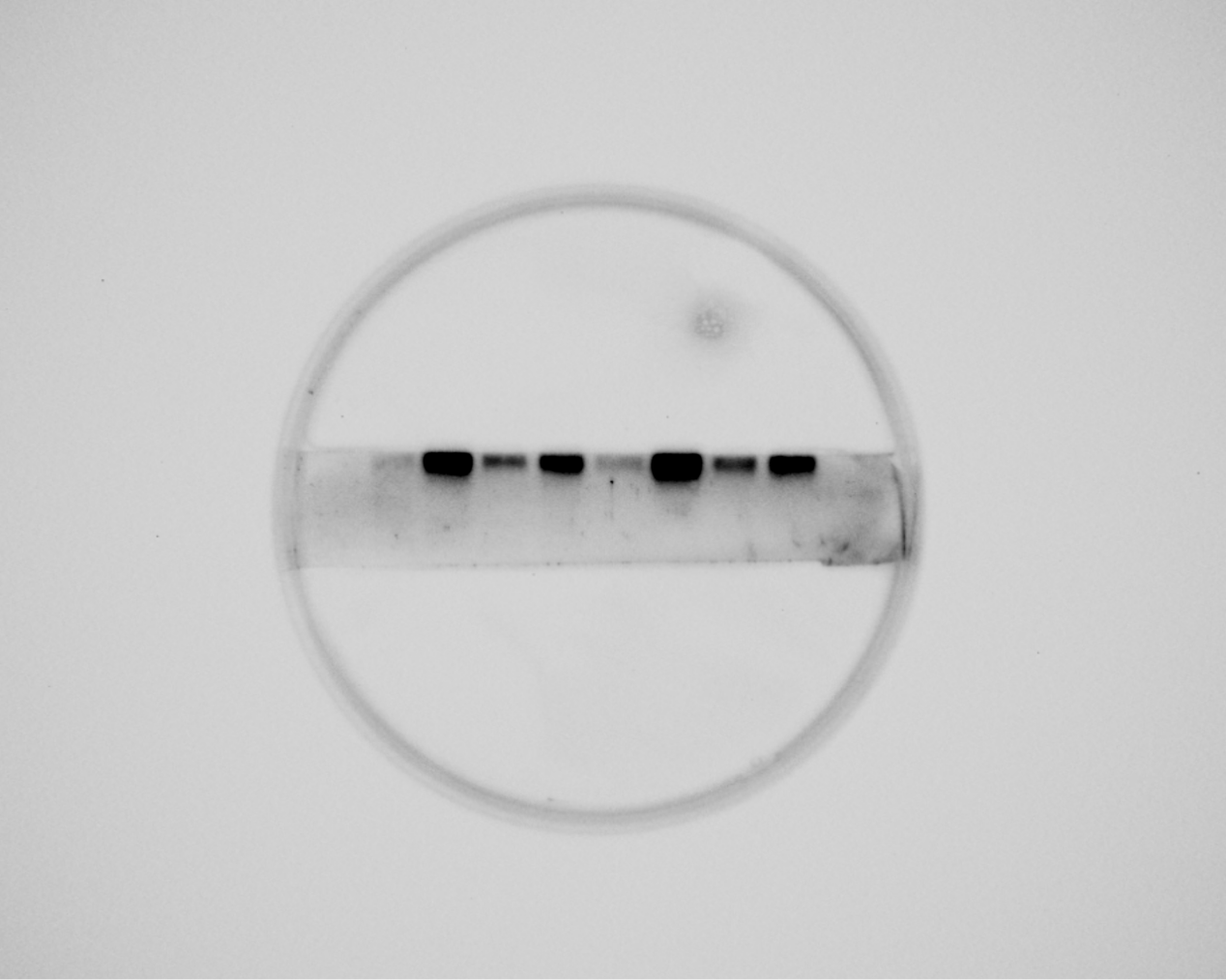


Casepase-3

Act

db/db

db/m

db/db

Act

Cap

db/m

Cap


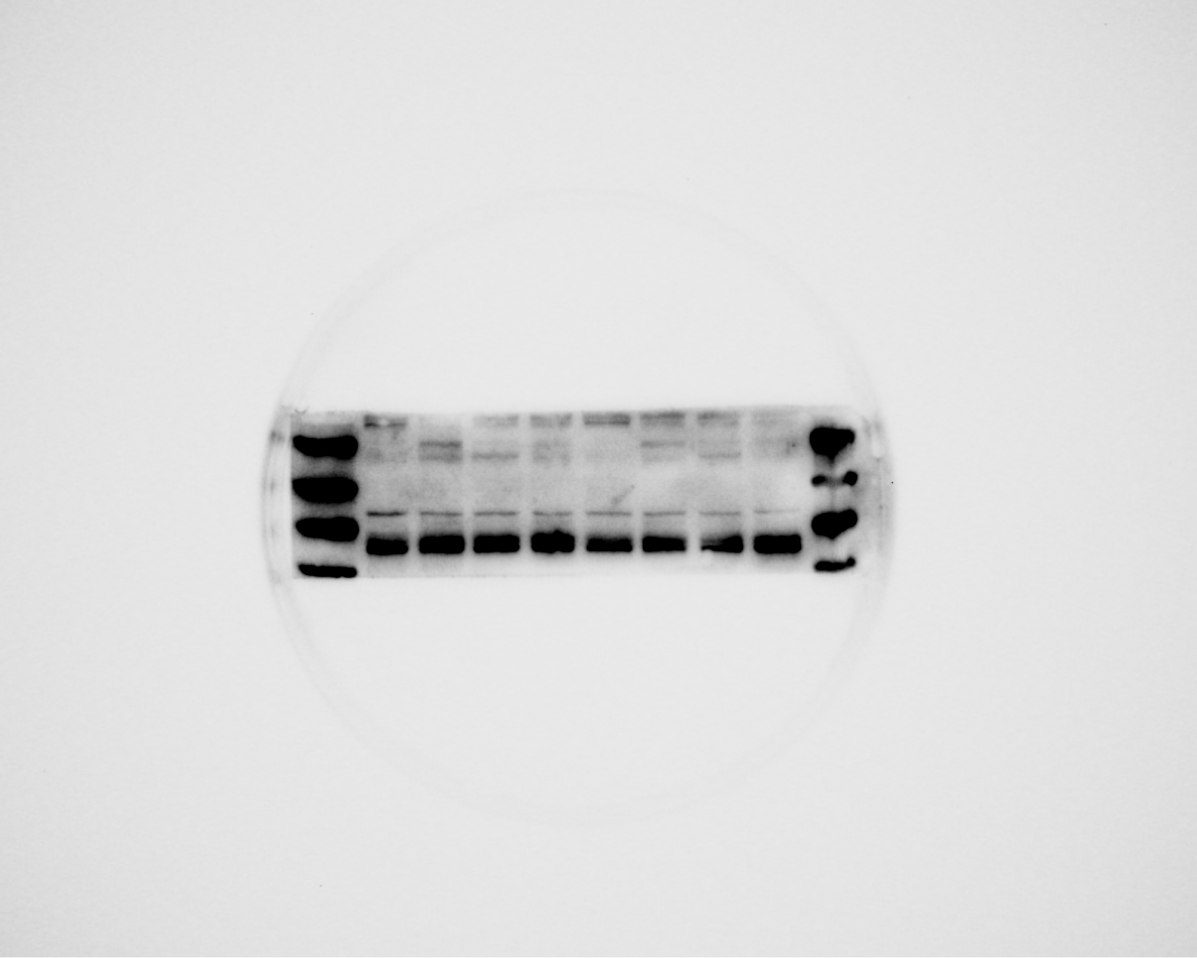


GAPDH

db/m

db/db

Act

Cap

Act

db/m

db/db

Cap


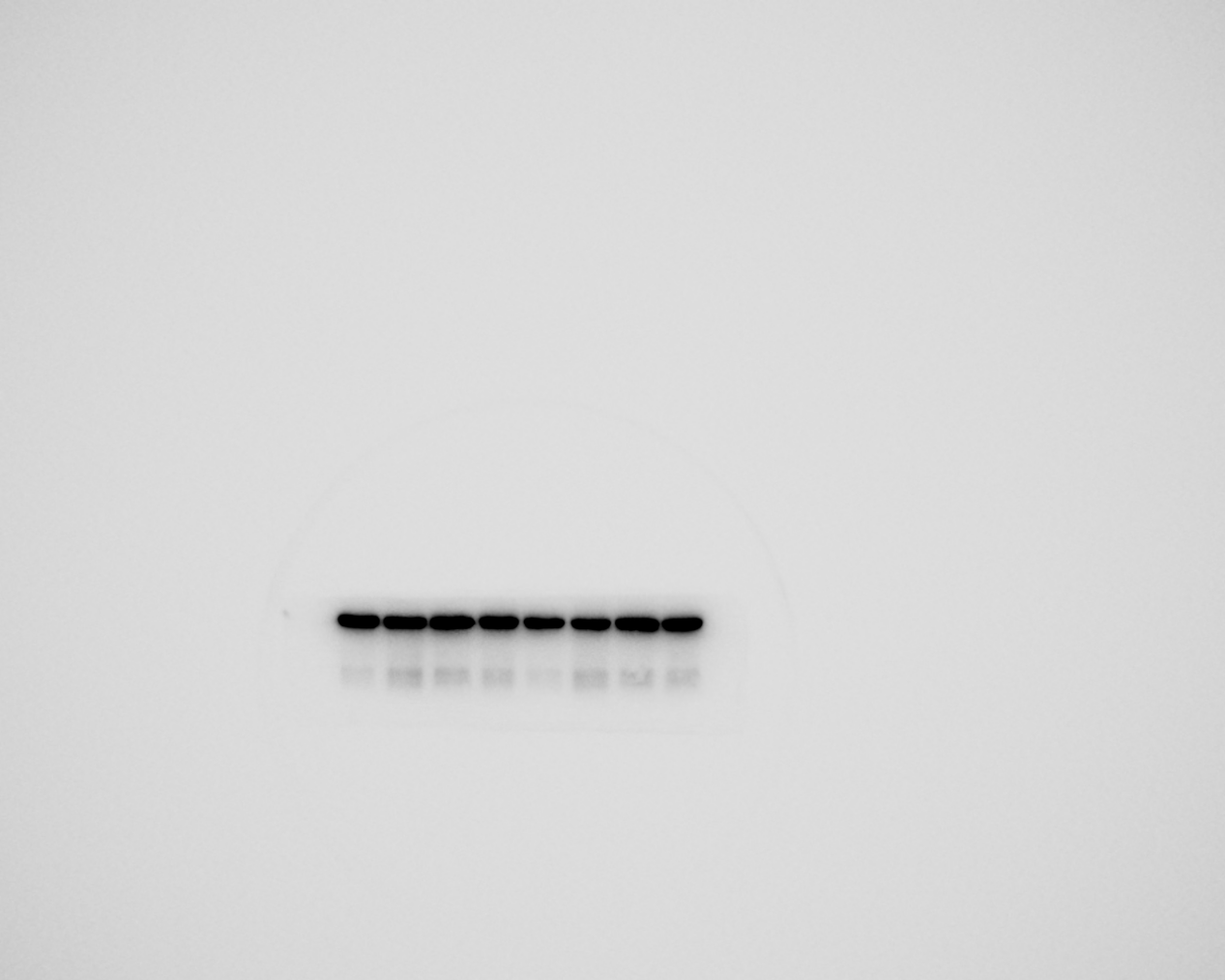


**Figure 4**. Effects of Act on renal tissue and podocytes. The Bcl-2, Bax, and cleaved caspase-3 expression in mice kidney tissue. The images are typical and representative.

Gels/blots are used in figure 5

p-AKT t-AKT

db/db

db/m

Act

Cap

db/m

db/db

Act

Cap

Cap

Act

db/db

db/m


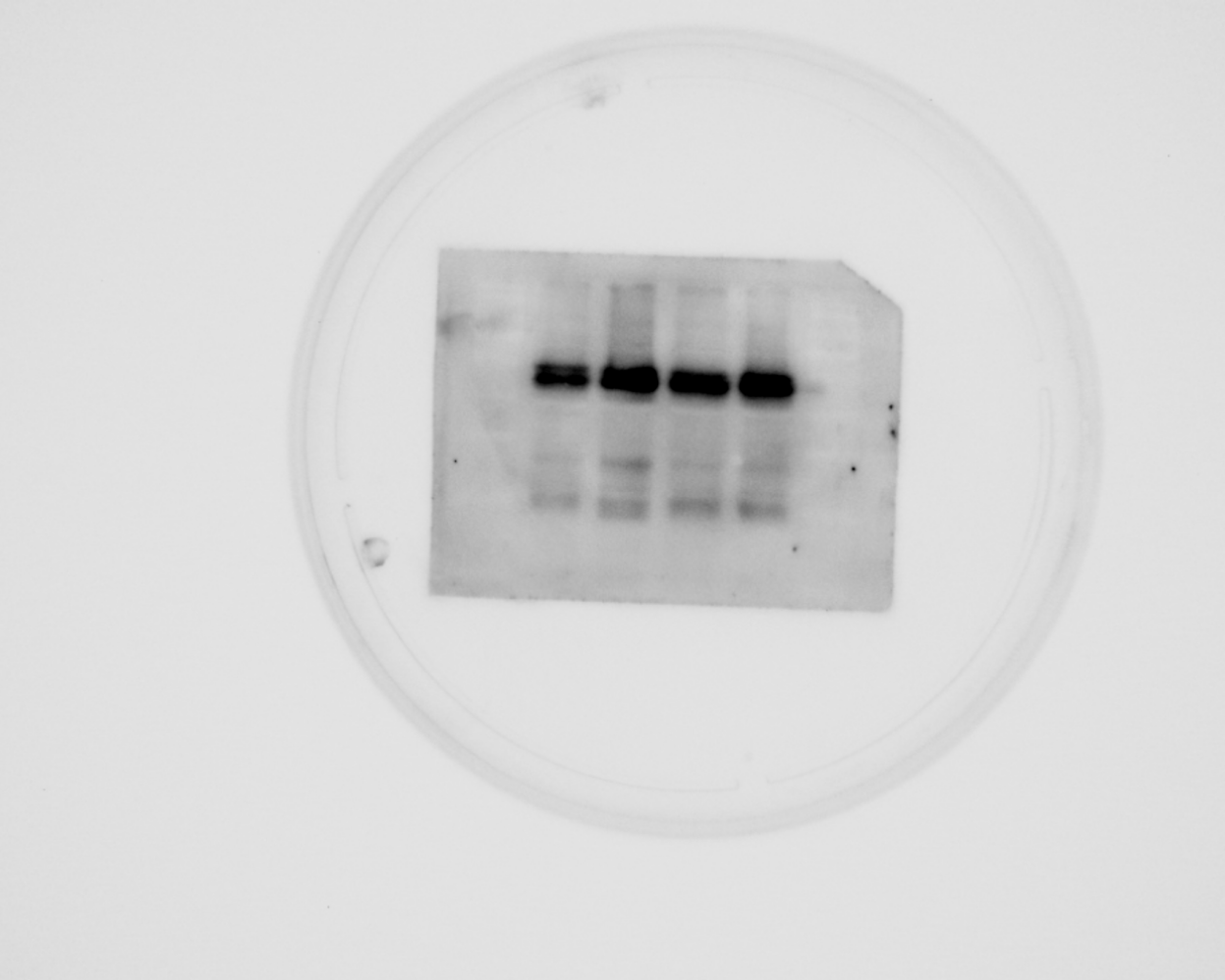

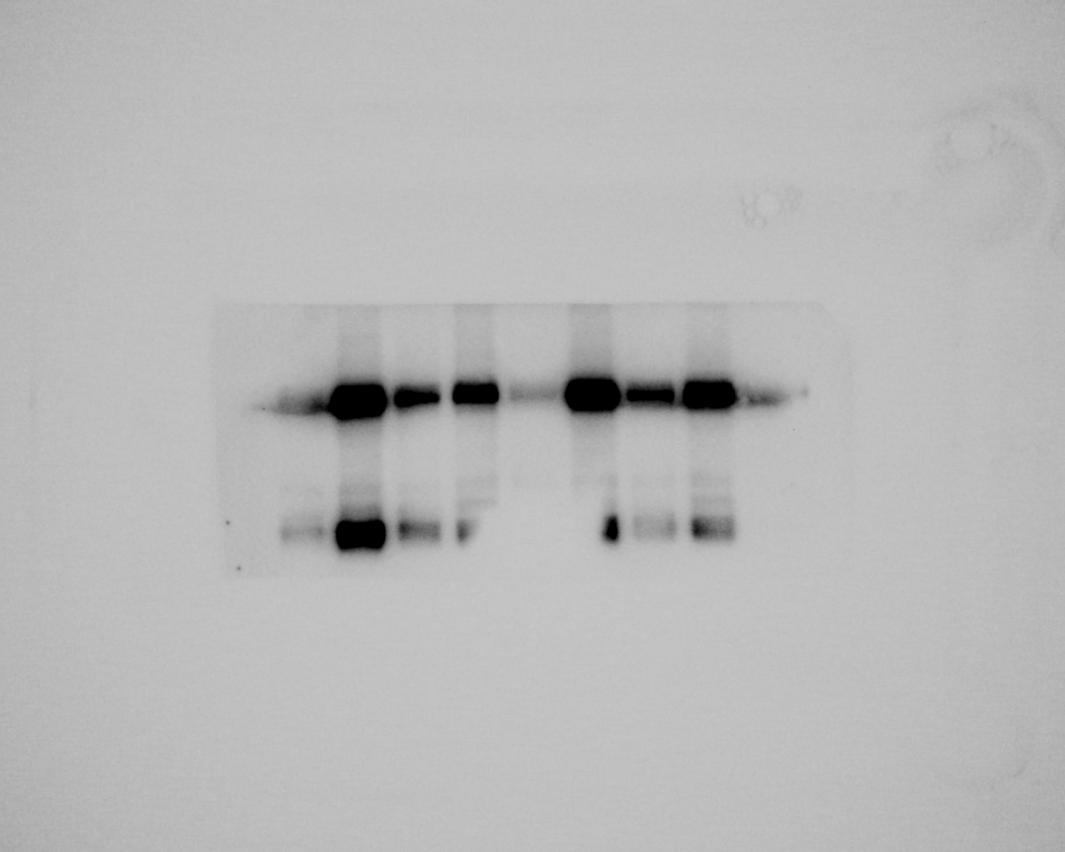


P-GSK-3β (ser-9) t-GSK-3β (ser-9)

Cap

Act

db/db

db/m

db/m

db/db

Act

Cap

Cap

Act

db/db

db/m

Cap

Act

db/db

db/m


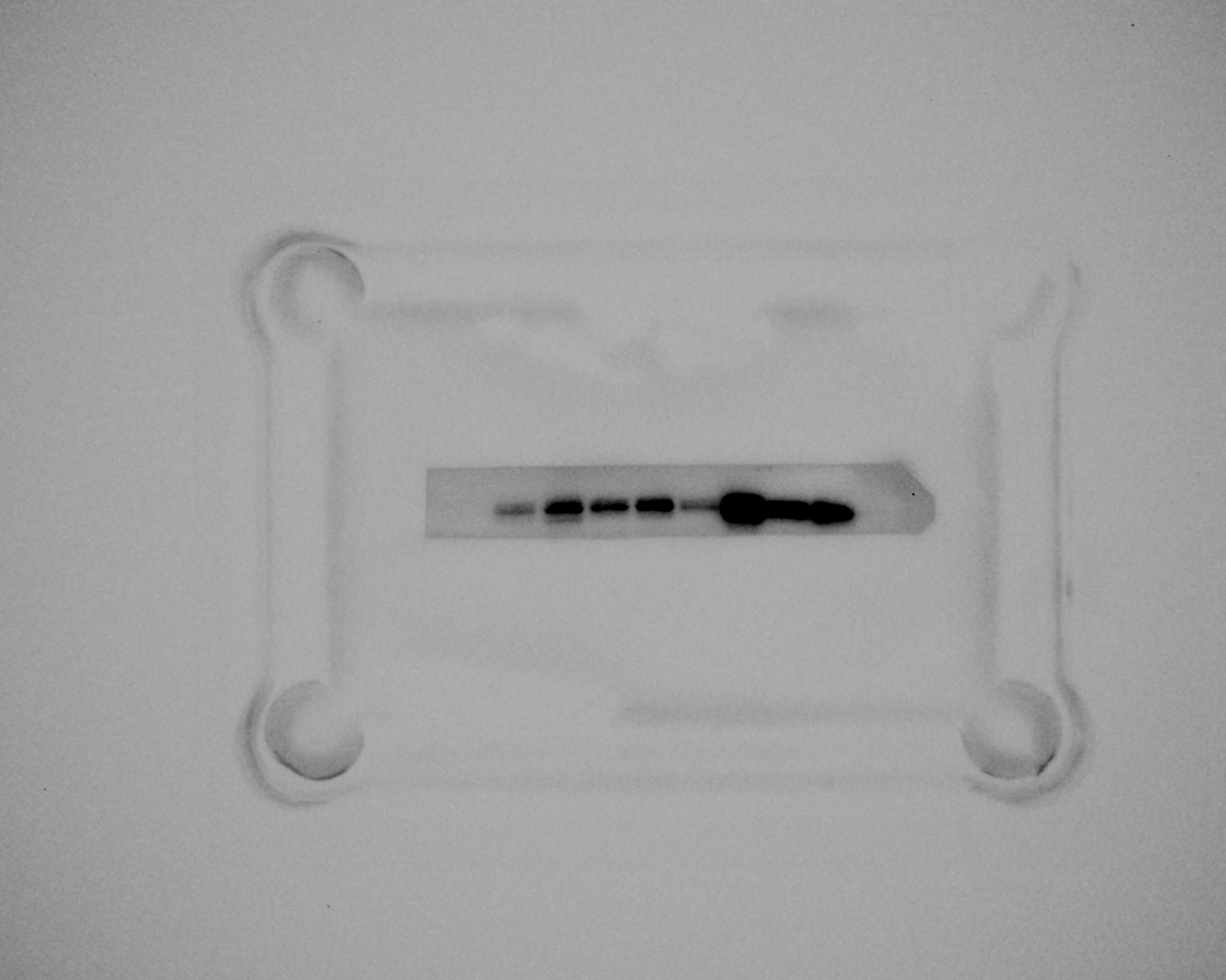

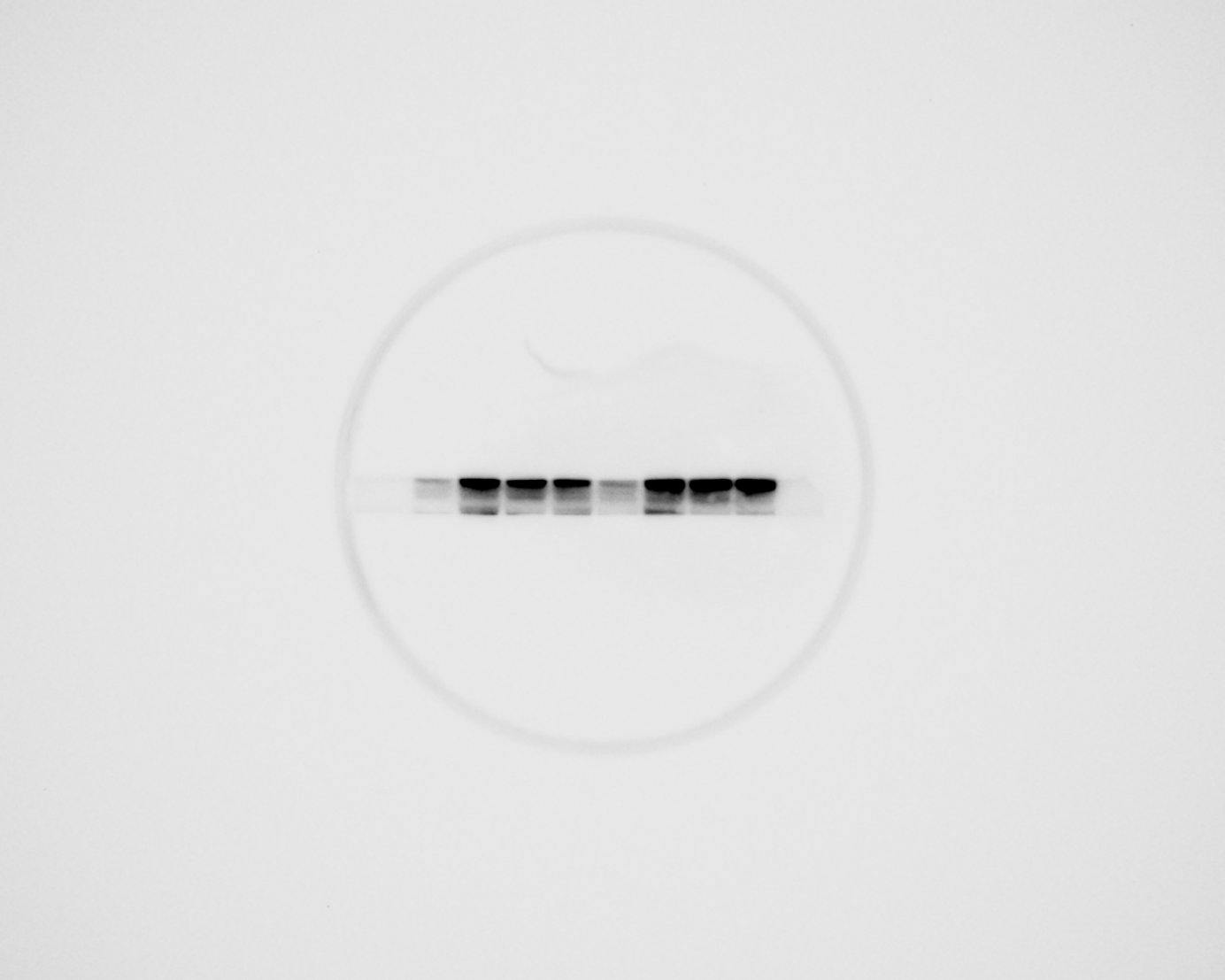


GAPDH

db/db

Act

Cap

Cap

Act

db/m

db/db

db/m


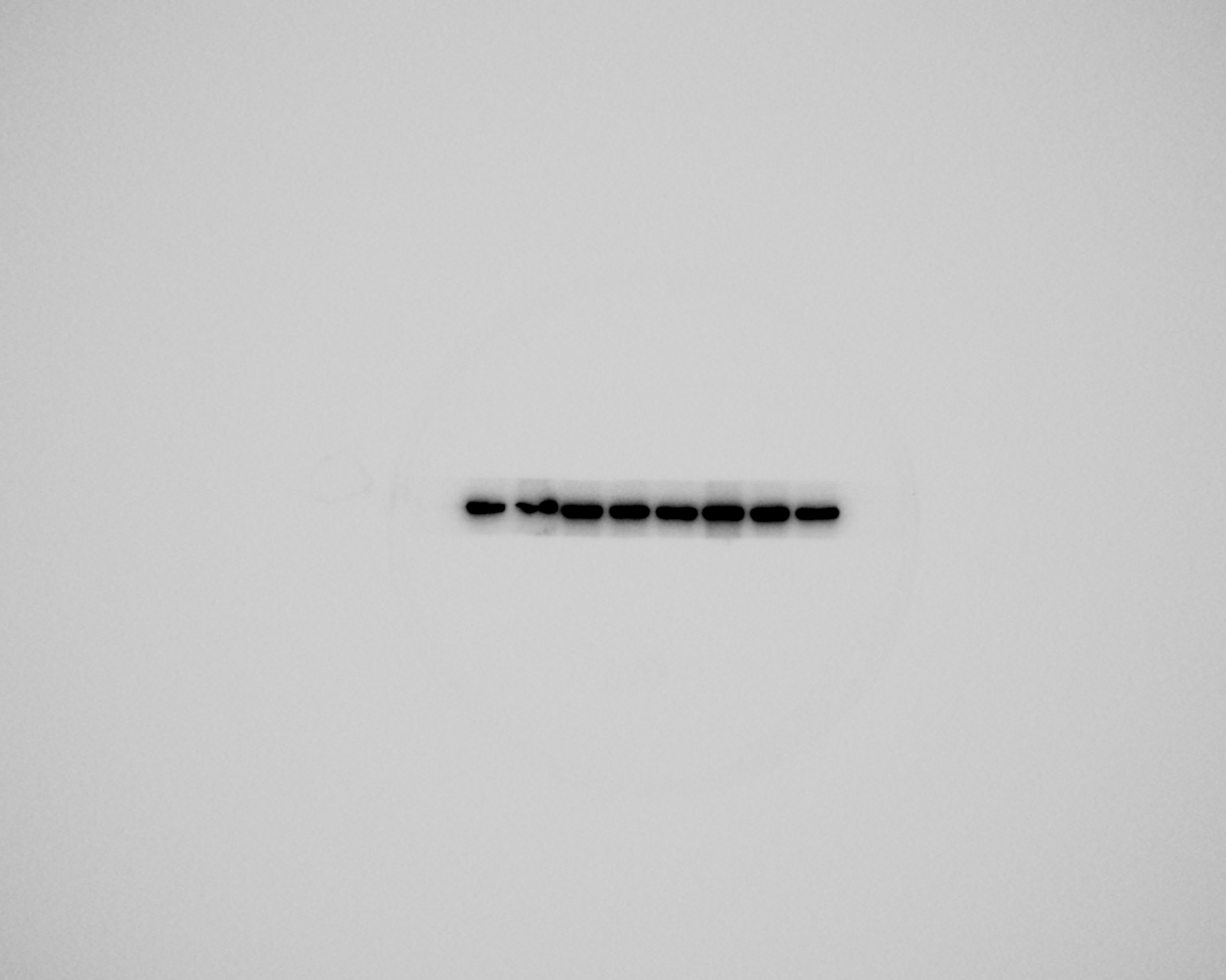


**Figure 5**. The protective effect of Act on podocytes may [correlated with](javascript:;) the AKT/GSK-3β signaling pathway inhibitory. Western blot consequences of p-AKT, p-GSK-3β in mice kidney tissues. The images are typical and representative.

Note:

1. All the above pictures are typical and representative.
2. Since the PVDF membrane was cut according to the molecular weight and incubated to the corresponding primary antibody during the western blot experiment, the gels/blots images provided in the supplementary material of western bolt figures are complete.
3. Gel/blot images exposure showing high contrast may be due to different devices, we did not manually adjust the exposure of the gel/blot image.
